# Investigation of Genetic Relatedness of *Brucella* Strains in Countries Along the Silk Road

**DOI:** 10.3389/fvets.2020.539444

**Published:** 2021-01-07

**Authors:** Zhiguo Liu, Chengling Wang, Kongjiao Wei, Zhongzhi Zhao, Miao Wang, Dan Li, Heng Wang, Qiang Wei, Zhenjun Li

**Affiliations:** ^1^National Institute for Communicable Disease Control and Prevention, Beijing, China; ^2^Qinghai Institute for Endemic Diseases Prevention and Control, Xining, China; ^3^Ulanqab Center for Endemic Disease Control and Prevention, Ulanqab, China; ^4^Hangzhou Center for Disease Control and Prevention, Hangzhou, China; ^5^Chinese Center for Disease Control and Prevention, Beijing, China

**Keywords:** *Brucella spp*., silk road, species/biovar, host lineages, geographic origin, genetic relatedness

## Abstract

In this study, MLVA (multiple-locus variable-number tandem repeat analysis) genotype data of *Brucella* strains from 11 countries along the Silk Road were downloaded from the MLVAbank. MLVA data of strains were applied to the constructed Minimum Spanning Tree to explore the species/biovars distribution, geographic origins, and genetic relationships of the strains analyzed. Moreover, whole-genome sequencing–single-nucleotide polymorphism (WGS-SNP) phylogenetic analysis of the genome of *Brucella melitensis* strains from GenBank was performed to discriminate the relatedness of strains further and investigate the transmission pattern of *B. melitensis* brucellosis. A total of 1,503 *Brucella* strains were analyzed in this study: 431 *Brucella abortus* strains (29.8%), 1,009 *B. melitensis* strains (65.7%), and 63 *Brucella suis* strains (4.5%). *B. melitensis* biovar 3 was the dominant species and was shown to be widespread in all of the examined regions, suggesting that the prevention and surveillance of the *B. melitensis* population are a main challenge in these countries. A wide host spectrum was observed for this *Brucella* population; many animal reservoirs are a potential reason for the continuous brucellosis circulation in these countries. Although the *B. abortus* strains from the examined regions had common geographic origins, only a few shared genotypes were observed in different countries. These data revealed that the majority *of B. abortus* strains were spreading within the national borders. However, the *B. melitensis* strains from Italy originated from a Western Mediterranean lineage; strains from the other 10 countries originated from Eastern Mediterranean lineage, and this lineage was shared by strains from three to nine different countries, suggesting that the introduction and reintroduction of the disease in the 10 countries might have occurred in the past. Furthermore, the most shared MLVA-16 genotypes were formed in the *B. melitensis* strains from China, Kazakhstan, and Turkey, suggesting that the introduction and trade in sheep and goats have occurred frequently in these countries. WGS-SNP analysis showed that the *B. melitensis* in this study originated from the Malta (Italy) region. According to their territorial affiliation between four clade strains from these countries in genotype B, the absence of a clear differentiation suggests that strains continuously expand and spread in countries along with Silk Road. Active exchange and trade of animals (sheep and goats) among these countries are reasonable explanations. *B. suis* strains from different nations showed unique geographic origins and epidemiological characteristics. Therefore, there is an urgent need for the control of transfer and trade of infected sheep (goats) in countries along the Silk Road, namely, the strengthening of the entry–exit quarantine of sheep and goats and improvements in the diagnosis of animal brucellosis.

## Introduction

Brucellosis is a zoonotic infectious disease caused by members of the genus *Brucellae*, circulating in more than 170 countries, especially in the Mediterranean, Asia, and Central and South America (1). The disease is primarily contracted through direct or indirect contact with infected animals and their contaminants, although the consumption of contaminated animal products can also cause infection. Low fever, sweating, fatigue, and joint pain are the most common clinical manifestations of patients (2). Chronic brucellosis is challenging to cure, which affects the quality of life of patients and causes great family and socioeconomic burdens (3). In females, abortion and stillbirth are the main manifestations, whereas orchitis and sterility are common in males (4). Therefore, brucellosis not only has a significant impact on the husbandry and health of the human population, but is also closely related to many fields such as public safety, food hygiene, and foreign trade (5).

Although human brucellosis (HB) is one of the most common bacterial zoonotic diseases, its occurrence greatly differs between geographic areas. The highest annual incidences of HB per million of the population are observed in Syria and Mongolia (1); for instance, 39,838 human cases were reported in Syria in 2007. Mongolia has a high incidence of brucellosis in humans and animals due to livestock husbandry; livestock seroprevalence ranged from 0.5 to 1.8% in 2011 (6). Moreover, a total of 684,380 HB cases were reported in mainland China during 1950–2018, and the incidence of HB peaked in 2014 (4.32/100,000) (7). Kazakhstan is a hyperendemic area, considering the very high incidence rates in the human population and farm animals (1). From 1999 to 2016, 38,557 new cases of HB were recorded, with annual counts of cases ranging from 1,443 (2014) to 3,596 (2004) (8). In Pakistan, the high age-wise prevalence was recorded as 32.25% (98/304) by enzyme-linked immunosorbent assay (ELISA) (*P* < 0.05) in 21- to 30-year-old females (9). In India, the seroprevalence of brucellosis among cattle and goats was estimated to be 1.1 and 11.2%, respectively (10). Brucellosis is still reported from all types of farms and domestic livestock in Russia; between 1999 and 2009, the number of infected sites increased, ranging from 54 in 2001 to 112 in 2009 (11). The incidence of HB in Turkey is 26 per 100,000 (12), and 18,264 new brucellosis cases were reported in 2004 (13); moreover, pediatric brucellosis may constitute 20% to 30% of all brucellosis cases in the world (14). Previous research reported that 22 (5.1%) tested by Rose Bengal Test (RBT) and 58 (13.5%) tested using the blocking ELISA assay were positive for *Brucella* in wild boar hunted in the Campania region of Italy during 2016–2017 (15). Each year a relatively small number of cases are reported in Germany, but within the last 2 years, an increase of cases was observed (16). Many comprehensive brucellosis controls have been developed in the majority of countries, including testing-slaughter, restricted infected animal transfer, and vaccination of livestock. However, brucellosis is still an endemic zoonosis in these nations (17).

The Silk Road Economic Belt has had a profound effect on economic prosperity, cultural diversity, foreign trades, and many other characteristics of nations along the road; therefore, it might also provide opportunities for the spread of zoonotic diseases (18). Indeed, zoonotic diseases such as plague and anthrax are associated with commercial activities related to the Silk Road (19, 20), especially in countries along the line of the road. Indeed, these countries have high amounts of husbandry, and brucellosis circulates between humans and animals in Italy, Germany, Turkey, Iran, and Kazakhstan (21–25). Accordingly, there is the potential for transmission of brucellosis during processing of animals and trade of animal products. Currently, MLVA-16 genotyping of *Brucella* strains can be used for trace-back and trace-forward investigations, as well as the identification of the spreading route (26), and MLVA-11 is often used to trace back the geographic origin of strains (27). Moreover, whole-genome sequencing–single-nucleotide polymorphism (WGS-SNP) is likely to be a suitable tool for trace-back analysis of *Brucella melitensis*, as it is capable of suggesting the potential geographic origin of a given strain (16). Therefore, this study was conducted to better understand the epidemiological characteristics of brucellosis in countries along the Silk Road, provide valuable information for strengthening trade cooperation, and launch a corresponding strategy of prevention and control of brucellosis.

## Materials and Methods

### Data Source

Data pertaining to MLVA-16 of 1,503 *Brucella* from 11 related countries including China, Kazakhstan, Turkey, Germany, Italy, Iran, Israel, Lebanon, Mongolia, Syria, and India were obtained from the international MLVAbank database (http://microbesgenotyping.i2bc.paris-saclay.fr/databases) (2016–2018·V1.4.0), including the strain number (Named by the database), species/biovars, host types, isolated region, Panel 1 genotype, MLVA-11 genotype, and the number of tandem repeats at MLVA-16 loci. Moreover, strains from Kyrgyzstan and Tajikistan were not found in the international MLVAbank database.

### Strain Isolation and Identification

A total of 1,503 *Brucella* strains were collected from samples of patients and animals from 11 different countries. According to standard bacteriology approaches, all of the strains were isolated and identified (28). Conventional identification methods were used to assign the strains to biotypes. All of the strains were gram-negative, agglutinated with polyvalent brucellosis serum, had oxidase and catalase activity, synthesized urease, and were capable of growing in atmospheric conditions. Subsequently, BCSP-31 polymerase chain reaction (PCR) (29), AMOS-PCR (30), and/or *Brucella* ladder PCR (31) were applied to verify the results from the biotyping assays.

### DNA Preparation and Genotyping

A fresh single *Brucella* strain clone was picked up from a solid agar medium surface and then heat-inactivated at 80°C for 20 min. Subsequently, DNA was isolated using a phenol/chloroform extraction method and/or the QIAamp DNA Mini Kit (Qiagen, United States) according to the manufacturer's instructions. The MLVA-16 assay was performed as previously described (32, 33). Briefly, 16 loci were divided into three panels: Panel 1 (also called MLVA-8), Panel 2A, and Panel 2B. The combination of panels MLVA-8 and 2A was called MLVA-11, whereas the combination of all three panels (16 loci) was designated MLVA-16. The MLVA-11 panel allows for tracing the geographic origin of the strains analyzed, whereas the Panel 2B loci are highly discriminatory, and their combination with MLVA-11 was used for molecular epidemiology investigation. PCR was used to determine the number of repeats from each sample, and its products were purified and directly sequenced using an ABI Prism Big Dye Terminator. Size analysis of VNTR repeats was performed using GeneMapper 4.1 (Applied Biosystems).

### Data Processing and Analysis

A total of 1,503 *Brucella* strains were downloaded from the MLVAbank database, including 394 in China, 359 in Italy, 338 in Kazakhstan, 234 in Turkey, 58 in Germany, 37 in India, 28 in Lebanon, 20 in Syria, 14 in Israel, 12 in Mongolia, and 9 in Iran (Supplementary Table 1). The downloaded data were processed according to the species/biovars and genotypes of all *Brucella* strains using Excel 2016 software (Microsoft Corporation, Redmond, WA, USA). At the same time, the species/biovars and distribution regions of the strains were analyzed to investigate the epidemiological correlation of *Brucella* in the above countries. Subsequently, the Minimum Spanning Tree (MST) of *Brucella abortus* strains, *B. melitensis* strains, and *Brucella suis* strains were constructed using the BioNumerics 7.6 software based on the MLVA-11 data, and the genotype distribution and geographical origin of the strains were analyzed. The BioNumerics 7.6 software (Applied Maths, Sint-Martens-Latem, Belgium) based on unweighted pair group method using arithmetic averages was applied to construct the MST of *B. abortus* strains, *B. melitensis* strains, and *B. suis* strains based on MLVA-16 data and to explore their genetic relatedness. Moreover, the WGS-SNP phylogenetic analysis (34) was performed in 39 *B. melitensis* from 14 countries along the Silk Road (Supplementary Table 2) to explore further the genetic relatedness of strains and the transmission pattern of brucellosis in these nations.

## Results

### Species/Biovar Distribution Characteristics of *Brucella* Strains

**Figure 1:**
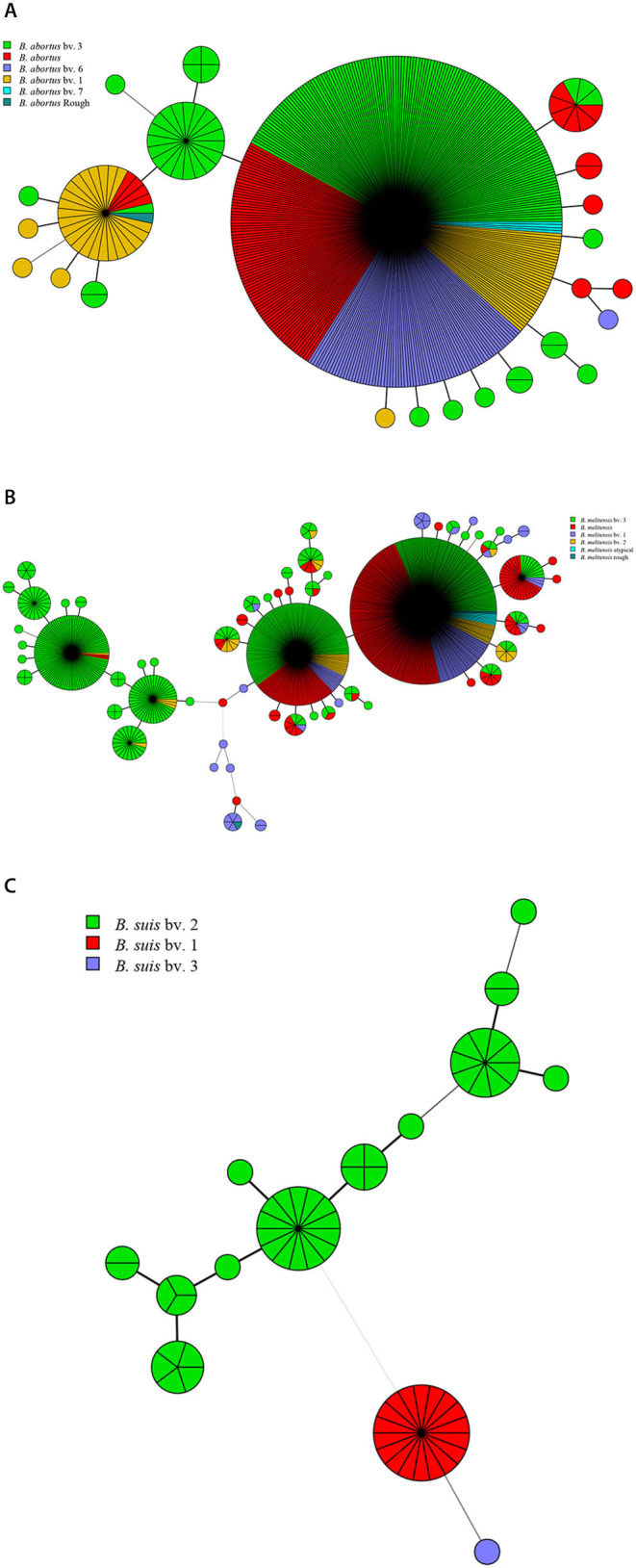
Species/biovars distributed characteristics of *B. abortus* **(A)**, *B. melitensis* **(B)**, and *B. suis* **(C)**. Color coding according to species/biovar; circle size depicts the number of strains.

Among the 1,503 strains of *Brucella*, 431 (28.7%) were *B. abortus*, 1,009 (67.1%) were *B. melitensis*, and 63 (4.2%) were *B. suis* (Table 1). *B. abortus* strains included four biovars: 63 in biovar 1, 188 in biovar 3, 79 in biovar 6, four in biovar 7, one in rough strain, and another 98 strains have not been identified (Figure 1A). Among the *B. melitensis* strains, 99 strains in biovar 1, 50 strains in biovar 2, 532 strains in biovar 3, five strains in rough, and nine strains were atypical, and 313 strains of unknown biovars were observed (Figure 1B). Among the 63 *B. suis* strains, 18 were in biovar 1, 44 in biovar 2, and one in biovar 3 (Figure 1C). All three *Brucella* species were observed in China, Germany, and Italy (Table 1). Both *B. abortus* and *B. melitensis* were circulating in the remaining seven countries. Five different biotypes were found in *B. melitensis* species; five biotypes were observed in *B. abortus* species, whereas three biotypes were recorded in *B. suis* species. *B. melitensis* was the dominant population in all countries examined, and *B. abortus* biovar 3 and *B. melitensis* biovar 3 were the dominant species in countries along the Silk Road (Table 1). *B. suis* biovar 1 was found exclusively in Chinese strains, and *B. suis* biovar 2 was observed in Germany and Italy (Table 1).

**Table 1:** Species distribution characteristics of *Brucella* strains in countries along the Silk Road.

**Species-biovars**	**China**	**Kazakhstan**	**Turkey**	**Germany**	**Italy**	**Iran**	**Israel**	**Lebanon**	**Mongolia**	**Syria**	**India**	**Total**
*B. abortus^†^*	—	98	—	—	—	—	—	—	—	—	—	98
*B. abortus bv.1*	5	36	—	2	7	—	—	—	—	—	13	63
*B. abortus bv.3*	65	1	1	3	116	—	—	—	—	—	—	188
*B. abortus bv.6*	—	78	—	—	—	—	—	—		1	—	79
*B. abortus bv.7*	—	—	1	—	—	—	—	—	3	—	—	4
*B. abortus Rough*	—	—	—	—	—	—	—	—	—	—	1	1
*B. melitensis^†^*	119	107	32	—	2	7	—	27	—	16	2	313
*B. melitensis bv.1*	52	14	7	4	4	—	10	—	5	—	3	99
*B. melitensis bv.2*	12	1	18	7	3	—	1	1	—	3	4	50
*B. melitensis bv.3*	123	3	175	8	217	2	2	—	—	—	4	534
*B. melitensis Rough*	—	—	—	—	—	—	1	—	4	—	—	5
*B. melitensis atypical*	—	—	—	—	—	—	—	—	—	—	9	9
*B. suis bv.1*	18	—	—	—	—	—	—	—	—	—	—	18
*B. suis bv.2*	—	—	—	34	10	—	—	—	—	—	—	44
*B. suis bv.3*	—	—	—	—	—	—	—	—	—	—	1	1
Total	394	338	234	58	359	9	14	28	12	20	37	1,503

† *unknown in biotypes; —, no data*.

### Host Lineages Profiles of *Brucella* Strains

**Figure 2:**
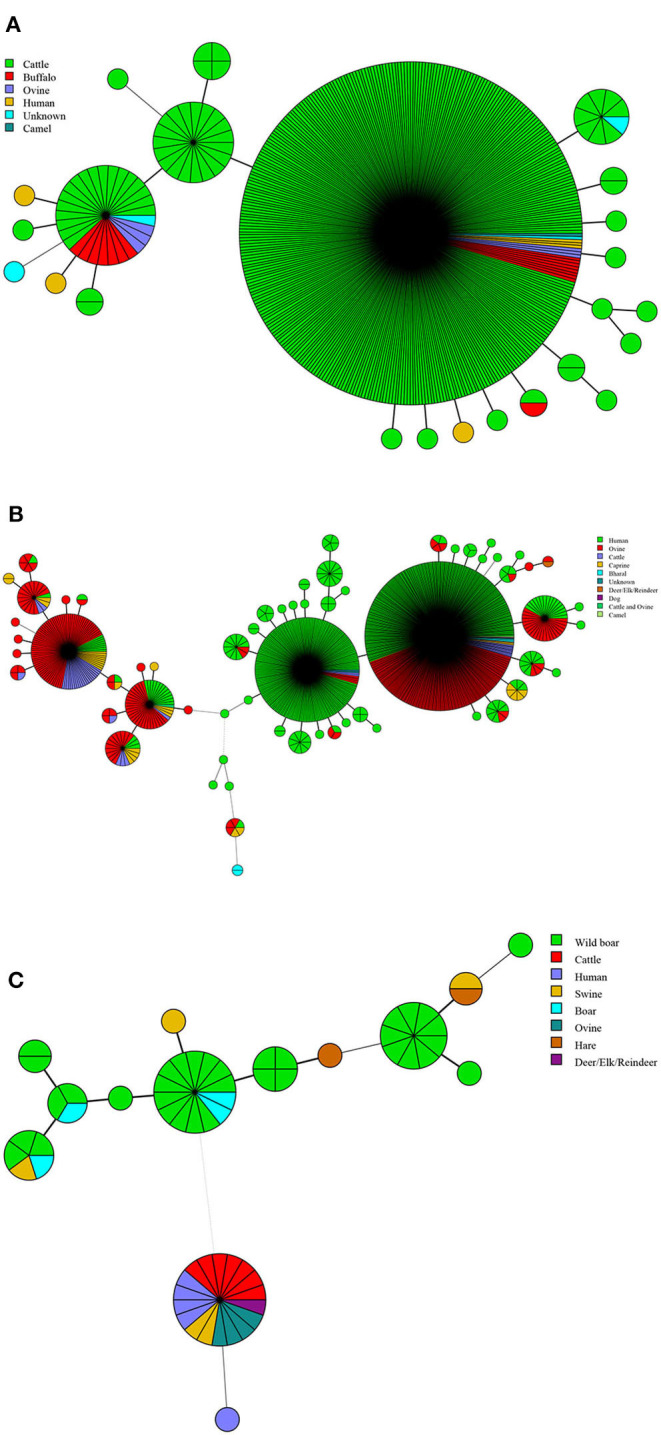
Host lineages profile of *B. abortus* **(A)**, *B. melitensis* **(B)**, and *B. suis* strains **(C)**. Color-coding according to hosts types; circle size depicts the number of strains.

In this study, a total of 13 kinds of hosts were observed in all of the countries analyzed, including humans, cattle, sheep, camels, buffalo, goats, bharals, dogs, deer, swine, boars, hares, and wild swine, of which 583 strains were found in humans, 448 in cattle, and 363 in sheep (Table 2). The hosts of *B. abortus* strains included cattle, sheep, camels, buffalo, and humans, of which 92.0% of strains were collected from cattle (Figure 2A). The hosts of *B. melitensis* strains included goats, sheep, cattle, deer, bharals, camels, humans, and dogs. Remarkably, approximately 56.8% (573/1,009) of strains were from humans, and this host was distributed widely in all of the countries; moreover, 35.3% (353/1,009) of the strains were obtained from sheep (Figure 2B, Table 2). Deer, humans, sheep, swine, boars, hares, and wild swine were common hosts from isolated *B. suis* strains, and 55.6% (35/63) of the strains from Germany and Italy were found in wild swine (Figure 2C). However, humans, cattle, and sheep were the main hosts of the *B. suis* strains in China (Table 2). The most extensive hosts of *Brucella* in this study were observed in China, followed by Italy, Kazakhstan, and India (Table 2).

**Table 2:** Host profiles of *Brucella* strains in countries along the Silk Road.

**Host**	**China**	**Kazakhstan**	**Turkey**	**Germany**	**Italy**	**Iran**	**Israel**	**Lebanon**	**Mongolia**	**Syria**	**India**
*B. abortus*	Cattle, humans, ovines	Cattle, camels	Humans	Cattle	Buffalo, cattle, ovines	—	—	—	Cattle	Cattle	Buffalo, human, cattle
*B. melitensis*	Humans, ovines, deer, bharals, camels, caprines, cattle	Cattle, dogs, humans, ovine	Humans	Humans	Caprines, cattle, humans, ovines	Humans	Ovine, human, cattle	Human	Human	Human, ovine	Ovine, human, caprine
*B. suis*	Deer, humans, ovines, swine	—	—	Boars, hares, swine	Wild boars	—	—	—	—	—	Human

—*, no data*.

### Geographic Origin and Genetic Relatedness of *B. abortus* Strains

**Figure 3:**
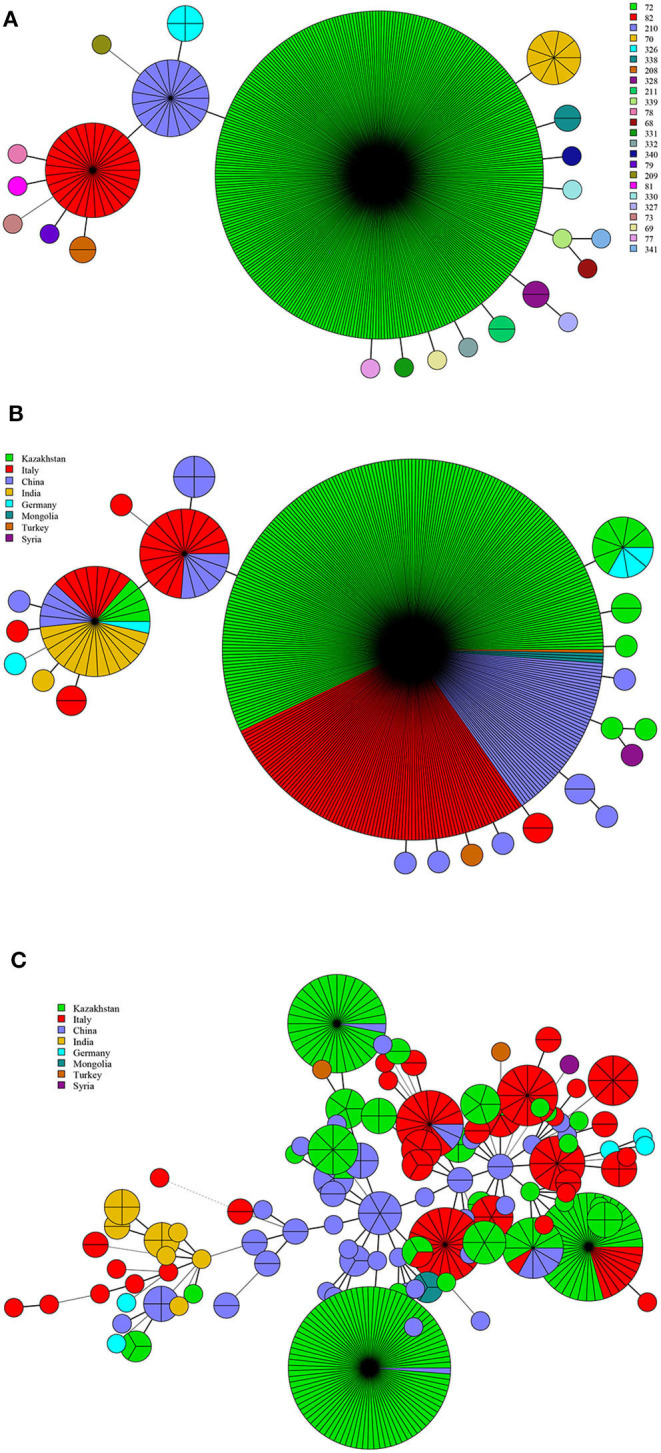
MLVA-11 genotype **(A)**, geographic origin **(B)**, and genetic relatedness characteristics **(C)** of *B. abortus* strains. (Color-coding according to MLVA-11 genotypes **(A,B)** and MLVA-16 genotypes (countries, **C**); circle size depicts the number of strains).

There were 24 MLVA-11 genotypes among *B. abortus* strains (72, 82, 201, and 70 were the most common genotypes). Of these, genotype 72 was overwhelming, accounting for 80.5% (347/431) (Figure 3A). Genotype 72 was shared by strains from Kazakhstan, China, Italy, Mongolia, and Turkey. Genotype 210 was shared by Italian and Chinese strains, whereas genotype 82 was shared by strains from Kazakhstan, German, Italy, China, and India (Figure 3B). MST analysis of *B. abortus* strains revealed that some MLVA-16 shared genotypes were found in strains from Kazakhstan, Italy, and China. Moreover, in Kazakhstan and China, Kazakhstan and Italy, and Kazakhstan, Italy, and China, a limited number of strains consisted of shared genotypes. In contrast, no shared genotype was found in other countries (Figure 3C).

### Geographic Origins and Genetic Relatedness of *B. melitensis* Strains

**Figure 4:**
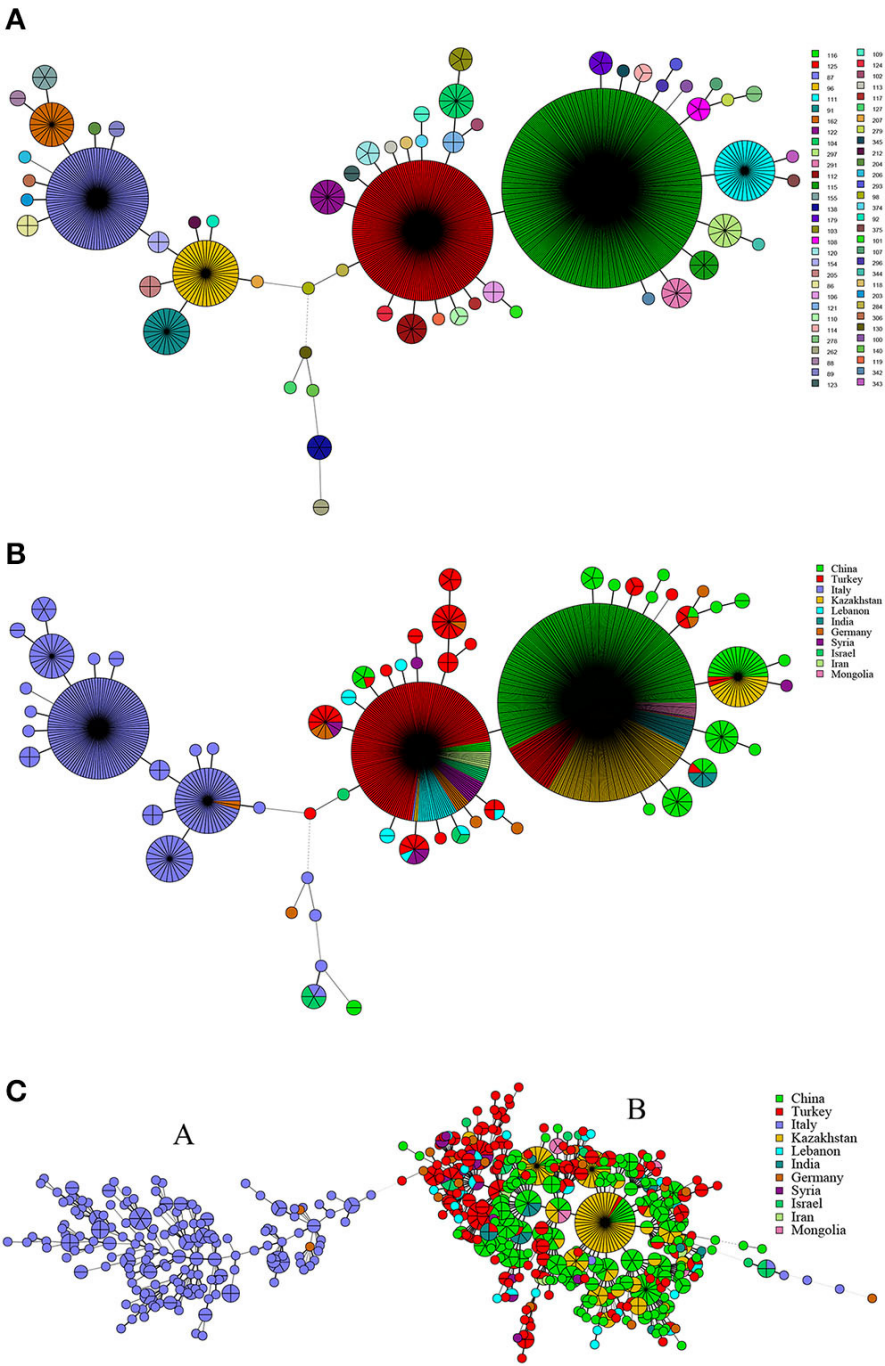
MLVA-11 genotype **(A)**, geographic origin **(B)**, and genetic relatedness characteristics **(C)** of *B. melitensis* strains. (Color-coding according to MLVA-11 genotypes **(A,B)** and MLVA-16 genotypes (countries, **C**); circle size depicts the number of strains).

There were 62 MLVA-11 genotypes in 1,009 strains of *B. melitensis*, of which 116, 125, 87, 96, 111, and 91 were the most common genotypes (Figure 4A). Among these, genotype 116 was overwhelmingly dominant, accounting for 40.9% (413/1,009), whereas genotypes 125, 87, 96, 111, and 91 accounted for 20.0% (202/1,009), 10.6% (107/1,009), 4.6% (45/1,009), 3.8% (38/1,009), and 2.2% (22/1,009), respectively. Based on the geographic origin, *B. melitensis* genotypes 116, 125, and 111 belong to an Eastern Mediterranean lineage, whereas genotypes 87, 96, 91, and 162 belong to the Western Mediterranean lineage. Genotypes 116, 125, and 111 were shared by strains from 10 countries, except Italy (Figure 4B). Genotype 116 was shared by strains from China, Germany, India, Iran, Kazakhstan, Lebanon, Mongolia, Syria, and Turkey, whereas type 125 was shared by strains from nine countries, namely, China, Germany, Iran, Israel, Italy, Kazakhstan, Lebanon, Syria, and Turkey. Otherwise, genotype 111 was observed in China, Kazakhstan, and Turkey. However, genotypes 87, 96, 91, and 162 were found almost exclusively in the Italian strains, and only two strains from Germany were represented in genotype 96 (Figure 4B). MST analysis based on MLVA-16 data of *B. melitensis* strains indicated that 1,009 strains of *B. melitensis* were clustered into two groups (A and B). The strains in Italy have formed an independent group A, whereas the other 10 countries' strains were clustered into group B (Figure 4C). Multiple shared genotypes in group B were observed in different countries, including China, Kazakhstan, and Turkey; China, Turkey, and Syria; China, Kazakhstan, and Mongolia; China and India; Turkey, Lebanon, and Syria; Turkey and Lebanon; Turkey and Syria; Turkey and India; and Turkey and Iran. The most shared genotypes were observed among the Chinese and Kazakhstan strains (Figure 4C).

### WGS-SNP Analysis of 39 *B. melitensis* Strains From 14 Countries Along the Silk Road

**Figure 5:**
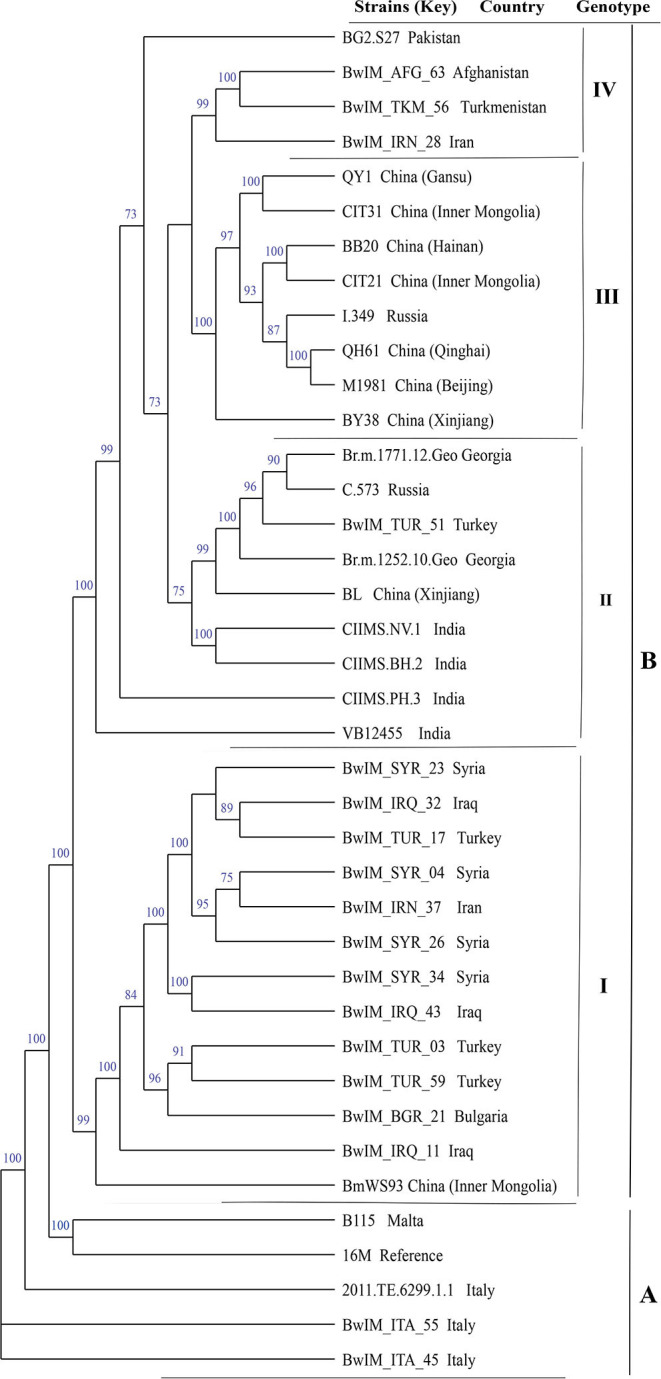
Whole-genome sequencing–single-nucleotide polymorphism phylogenetic analysis of 39 *B. melitensis* strains from 14 countries along the Silk Road.

Phylogenetic analysis based on WGS-SNPs revealed the geographic clustering profile of *B. melitensis* strains from these countries; 39 *B. melitensis* strains were divided into two groups (A and B). Group A included the strains from the Western Mediterranean Region (Italy and Malta) and located at the base of the tree. Group B included the strains from regions of Asia, covering a broad geographic area that could be subdivided into four clades: (I) Middle East (Iran, Iraq, Turkey, Syria), China, and Bulgaria; (II) Turkey, Russia, China, Georgia, and India; (III) China-Russia; (IV) Turkmenistan, Afghanistan, Iran, and Pakistan (Figure 5).

### Geographical Origin and Genetic Relatedness of *B. suis* Strains

**Figure 6:**
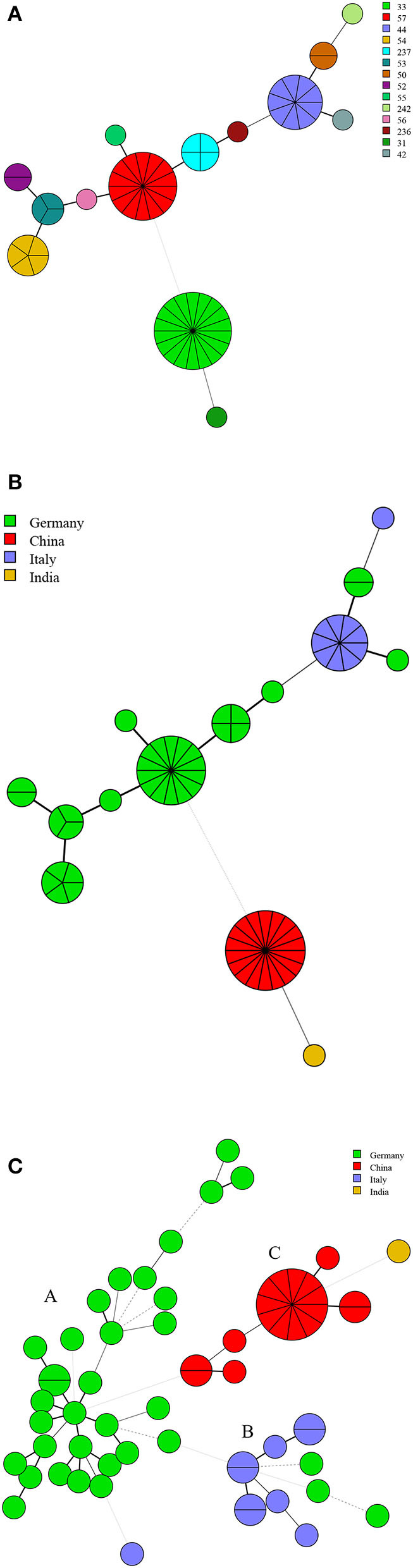
MLVA-11 genotype **(A)**, geographic origin **(B)**, and genetic relatedness characteristics **(C)** of *B. suis* strains. (Color-coding according to MLVA-11 genotypes **(A,B)** and MLVA-16 genotypes (countries, **C**); circle size depicts the number of strains).

There were 14 MLVA-11 genotypes in 63 strains of *B. suis*, and MLVA-11 genotypes 33, 57, and 44 were predominant, accounting for 28.6% (18/63), 22.2% (14/63), and 7.0% (9/63), respectively; the remaining 11 genotypes contained one to five strains from the respective country (Figure 6A). Genotypes 33, 31, 57, and 44 were unique to Chinese, India, German, and Italian strains, respectively (Figure 6B). MST analysis based on MLVA-16 data revealed that 63 strains of *B. suis* from Germany, Italy, China, and India formed three independent branches (A–C). The majority of strains from Germany clustered in branch A, and all Italy strains and a few German strains clustered in branch B. Strains from China and India (*n* = 1) were grouped in branch C. No shared MLVA-16 genotypes were found in strains from different countries (Figure 6C).

## Discussion

Brucellosis is prevalent in Italy, Germany, Turkey, Iran, Kazakhstan, China, and other countries, along with the Silk Road. Moreover, a considerable number of *Brucella* strains have been isolated, providing a basis for investigation of the genetic correlation characteristics among strains. In this study, *B. abortus, B. melitensis* strains, and *B. suis* strains accounted for 28.7% (431/1,503), 67.1% (1,009/1,503), and 4.2% (63/1,503) of the total, respectively. Moreover, *B. melitensis* biovar 3 accounted for 52.9% (534/1,009) of the total. The distribution characteristic of pathogenic bacteria population and *Brucella* biovars was similar to the previous study; *B. melitensis* is the most important zoonotic agent, followed by *B. abortus* and *B. suis* (35, 36). This correlates with the fact that the worldwide control of bovine brucellosis (due to *B. abortus*) has been achieved to a greater extent than the control of sheep and goat brucellosis (due to *B. melitensis*), these latter species being the most important domestic animals in many developing countries (17). *B. melitensis* strains were distributed widely in all of the countries examined; 56.8% (573/1,009) strains were obtained from humans, and 35.3% (353/1,009) were from sheep. These data indicated that *B. melitensis* strains are the predominant pathogen responsible for human and animal brucellosis in these countries. These findings suggest that identical or similar epidemiological characteristics of brucellosis might be found in countries along the Silk Road (37). Globally, despite the remarkable results achieved by the majority of industrialized countries, where bovine brucellosis has been eradicated or controlled, small ruminant brucellosis remains a problem in some of these countries (38). Our current study confirmed that *B. melitensis* is a latent “travel bacterium” that continuously spreads and expands from Northern China to Southern China (39). From 1970 to 2015, *B. melitensis* has been the pathogen isolated most frequently in human cases, accounting for more than 99% of *Brucella* spp. isolated from humans in Italy (40). Similarly, the species that causes the most frequent brucellosis and produces the most severe disease picture is *B. melitensis* in Turkey (13). Although 28.7% of total strains were of *B. abortus*, most of them were obtained from cattle, and its pathogenicity to humans was significantly lower than *B. melitensis* strains (17). Moreover, *B. suis* biovar 2 strains in this study were from a wild animal; *B. suis* biovar 1 and biovar 3 from humans and *B. suis* strains were observed in limited countries; fewer strains were isolated from humans, and the *B. suis* biovar 2 is not an essential pathogen for humans in contrast to *B. suis* biovars 1, 3, and 4 (17). Therefore, to solve the brucellosis problem in countries along the Silk Road, we should focus more on the *B. melitensis* strains than *B. abortus* and *B. suis* populations, and it is urgent to strengthen the detection and management of the infected sheep and goats in these countries.

In the present study, *B. abortus* strains and *B. melitensis* strains had a wide host spectrum, including many livestock and wild animals. Some shared genotypes were observed in different hosts among each species, indicating that these wild animals might become a source for reintroducing *Brucella* strains in domestic ruminants and humans (41). *B. abortus* and *B. melitensis* are the most regularly transmitted species between wild and domestic ungulates. They are most frequently associated with the conflicting needs of wildlife and agriculture and the risk of human disease (42). This confirmed the widespread existence and natural epidemic origin of brucellosis in countries along the Silk Road (43, 44), which poses severe challenges to the prevention and surveillance of brucellosis in these countries.

Based on the MLVA-11 analysis of *B. abortus* strains, genotypes 72, 201, 82, and 70 were more common; each of them was shared by strains from two to five countries, especially 72 being the predominant genotype and shared by strains from Kazakhstan, China, Italy, Mongolia, and Turkey. These findings demonstrate that the *B. abortus* strains from these countries have high homogeneity and common geographic origins (45, 46). MST analysis of *B. abortus* strains revealed that there were bovine brucellosis outbreaks in Kazakhstan, Italy, and China, but the number of cases of cross-infection among countries was limited (47). This was not only closely related to the reduction in virulence of *B. abortus* strains, but also restricted by factors such as larger size in cattle, higher transportation costs, and trade policies.

Among the 1,009 *B. melitensis* population, genotypes 116, 125, and 111 originated from the Eastern Mediterranean lineage, and genotypes 87, 96, and 91 originated from Western Mediterranean lineage. These findings demonstrate that the *B. melitensis* strains from countries along the Silk Road have two different geographical origins. The strains from Italy originated from a Western Mediterranean lineage, and strains from the other 10 countries originated from an Eastern Mediterranean lineage. Moreover, genotypes 116, 125, and 111 were shared by strains from nine, nine, and three countries, respectively, indicating that the strains from these countries had the same geographical origin. This suggests that the introduction and reintroduction of the disease among the 10 countries might have occurred in the past (48, 49). Based on MST analysis revealed that *B. melitensis* strains clustered into two groups, with strains in Italy forming a relatively independent group A, whereas strains in the remaining 10 countries clustered into group B. In addition, there were at least 10 shared MLVA-16 genotypes in strains from group B. The most shared genotypes were formed in strains from China, Kazakhstan, and Turkey. It was previously suggested that *B. melitensis* strains in China, Turkey, and Kazakhstan were epidemiologically correlated, and the prevalence of *B. melitensis* brucellosis in these countries resulted from a common infectious source (16, 50, 51). The breeding and trade of small ruminants were the main economic source and industrial pillar of the vast agricultural and pastoral areas in Turkey, Kazakhstan, and China, and animal introduction and trade were extremely frequent in these countries. The results were consistent with those of previous studies that showed the majority of *B. melitensis* strains in Kazakhstan were genetically related to strains transmitted in China and were closely related to the long-term trading partnership between the two countries (52). Therefore, WGS-SNP analysis indicated that *B. melitensis* were originated from Malta (Italy) regions, strains from group B forming the four subclades, but do not have a clear differentiation according to their territorial affiliation, indicating the frequent penetration of the *B. melitensis* strains from one country to another (53). Active exchange and trade of animals (sheep and goats) among these countries could promote pathogen dissemination, and there was continuous expansion and spread in countries along with Silk Road. Recently, many infectious diseases have emerged, and most of their pathogens originated from zoonosis; some of the diseases spread worldwide by the international flow of goods, people, and animals (54). We suggest that implementing a larger scale of vaccine coverage of small ruminants would significantly reduce infection in livestock and humans. Moreover, restricting movement in positive sheep and goats among different countries and the regular disinfection of the farm environments could effectively reverse the trend of increasing brucellosis in countries along the Silk Road.

*B. suis* strains in Germany, China, Italy, and India represent a unique predominated genotype, respectively, suggesting that each of these strains had an exclusive geographic origin. MST analysis revealed that MLVA-16 shared genotypes were not found in different countries' strains, demonstrating that *B. suis* strains were only prevalent in their respective countries. This was possibly related to environmental adaptability and economic and social factors influencing hosts of *B. suis* strains. *B. suis* biovar 2 is largely restricted to continental Europe and is maintained in wild animal (*Suidae* and hares) populations (40). We suggest that enhanced surveillance regulation for wild animals in Europe is essential for the prevention of strain spill over into the livestock. Moreover, further genome comparison analysis of more *B. suis* strains from all over the world will increase our understanding of *B. suis* brucellosis epidemiology.

Our research has some limitations. Most of the data were derived from highly concentrated time and location, rather than from continuous time points and wide coverage areas, so they may only partly reflect the truth situation of brucellosis in the regions examined. Second, data on animals' epidemiology and movements in these countries were not available. A focus on risk analysis or statistical analysis of hosts from the spatiotemporal or species-location perspective is essential.

## Conclusion

In this study, the species distribution, host lineage profiles, geographical origin, and genetic relatedness and the epidemiological correlation of *Brucella* species in countries along the Silk Road were discussed. *B. abortus* was found to share the same geographic origin, but the transmission area was limited to their respective countries. However, *B. melitensis* strains were the dominant population in these countries and had two main geographic origins, and strains from the same geographic origin showed extensive gene sharing. *B. suis* strains from respective countries showed unique geographic origins and epidemiological characteristics. The surveillance and control of *B. melitensis* strains were identified as major problems among *Brucella* species in countries along the Silk Road. The control of its main host (sheep and goat) should be strengthened. Effective vaccination limits *Brucella* infection, restricts shedding, hampers transmission from animal to animal, and diminishes zoonosis risk (55). The corresponding regulations should be strictly implemented when introducing animals to prevent the spread of this species. Moreover, improving scientific research to achieve early diagnosis and implement joint prevention and control is recommended.

## Data Availability Statement

All of the data generated or analyzed during this study are included in this published article, and the supplementary information files will be freely available to any scientist wishing to use them for non-commercial purposes upon request via e-mail with the corresponding author.

## Author Contributions

ZhiL performed strain download, MLVA typing and cluster analysis. CW drafted the manuscript. KW, HW, MW, ZZ and DL conducted data processing. ZheL and ZhiL participated in design of the study and critically reviewed the manuscript and ZheL managed the project. QW revised the manuscript critically. All authors have read and approved the final manuscript.

## Conflict of Interest

The authors declare that the research was conducted in the absence of any commercial or financial relationships that could be construed as a potential conflict of interest.
