# Supplementary figures and images for: Investigation of Genetic Relatedness of Brucella Strains in Countries Along the Silk Road
Source: Front Vet Sci. 2021 Jan 7;7:539444. doi: 10.3389/fvets.2020.539444 (PMC7817895; doi:10.3389/fvets.2020.539444)

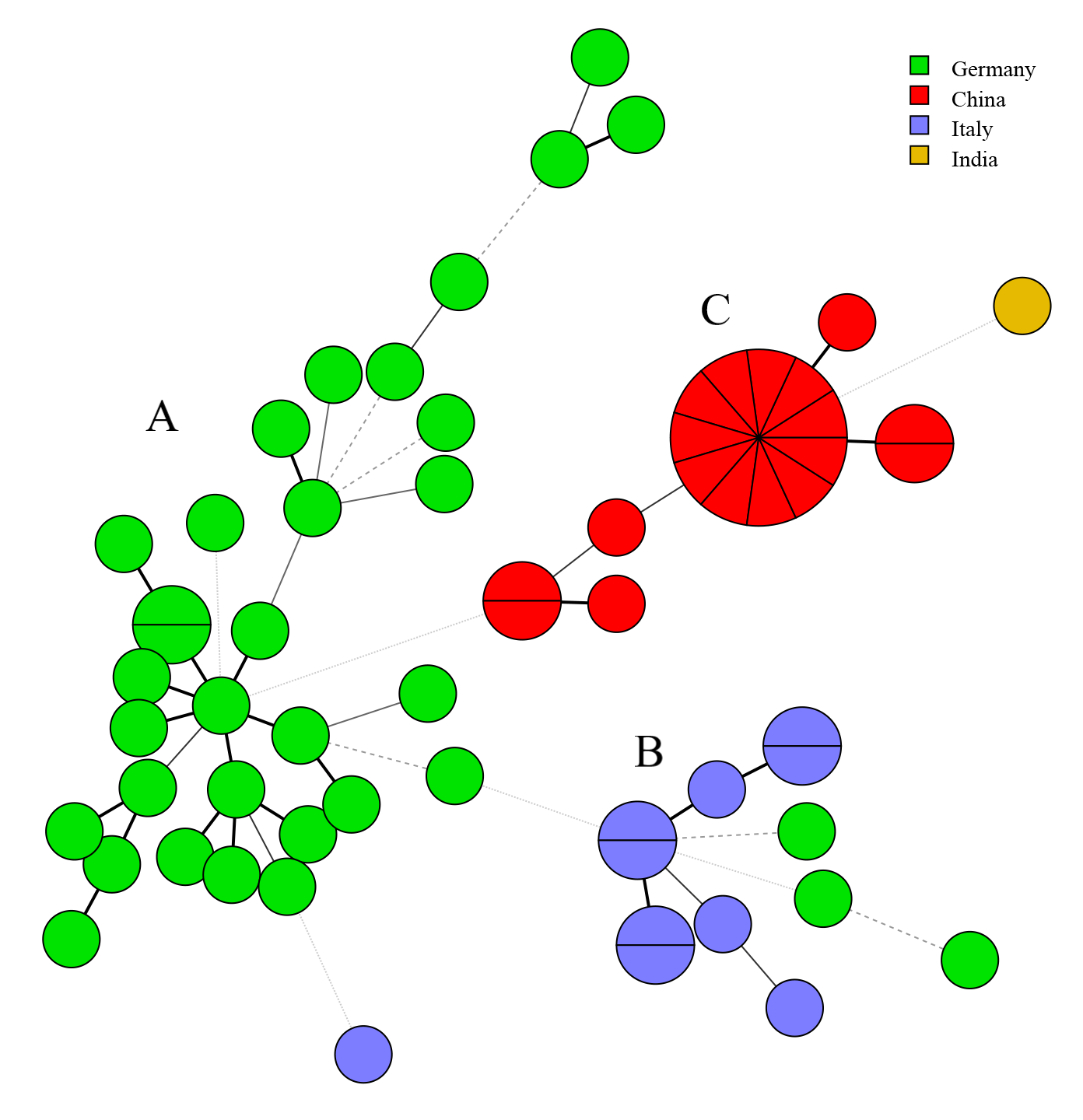

Supplement: Supplementary file 3 [file Image_1.TIF]
